# Characteristics of small airway disease in patients with HIV infection: insights from spirometry and impulse oscillometry

**DOI:** 10.1136/bmjresp-2025-003719

**Published:** 2026-04-15

**Authors:** Ilgim Vardaloglu, Buket Caliskaner Ozturk, Hazal Cansu Culpan, Bilgul Mete, Fehmi Tabak, Ersan Atahan

**Affiliations:** 1 Department of Chest Diseases, Istanbul University-Cerrahpasa Cerrahpasa Faculty of Medicine, Istanbul, Turkey; 2 Department of Public Health, Istanbul University-Cerrahpasa Cerrahpasa Faculty of Medicine, Istanbul, Turkey; 3 Department of Infectious Diseases and Clinical Microbiology, Istanbul University-Cerrahpasa Cerrahpasa Faculty of Medicine, Istanbul, Turkey

**Keywords:** Systemic disease and lungs, Viral infection, Respiratory Function Test

## Abstract

**Background:**

Airway diseases that are independent of smoking behaviour are frequent in people living with HIV (PLWH). Spirometry, the gold standard for diagnosing airway diseases, may not detect small airway disease (SAD) when forced expiratory volume in 1 s/forced vital capacity is normal. However, impulse oscillometry (IOS) can detect SAD even when the spirometry is normal. This study aims to evaluate characteristics of SAD in PLWH by using IOS and exploring the diagnostic performance of IOS measurements to detect SAD.

**Methods:**

This cross-sectional study included 127 PLWH on antiretroviral therapy without known airway disease. IOS was done first, followed by spirometry. Patients whose maximal mid-expiratory flow (MMEF) value was below 65% were defined as having spirometric SAD. Clinical characteristics and IOS measures were compared between those who had and those who did not have SAD. A receiver operating characteristic analysis was done to determine the diagnostic performance of IOS measures to diagnose spirometric SAD.

**Results:**

Mean age was 43.5±12.5 years and 60 patients were non-smokers. Spirometric SAD was observed in 34% of all patients. R5, R5–R20, AX and Fres were significantly higher in the patients who had spirometric SAD. Smoking history, duration of antiretroviral therapy and history of pneumonia were significantly associated with SAD. The optimal cut-off value for R5–R20 was 0.08 for SAD (sensitivity of 71.8% and specificity of 58.1%) and the optimal cut-off value for AX was 0.55 (sensitivity of 51.2% specificity of 91.7%).

**Conclusion:**

SAD is common in PLWH and IOS may serve as a supportive tool for the clinical assessment of small airway involvement.

**WHAT IS ALREADY KNOWN ON THIS TOPIC**

- Small airway disease (SAD) is an early finding in airway pathologies. People living with HIV (PLWH) are prone to chronic airway diseases which can be detected by impulse oscillometry (IOS).

**WHAT THIS STUDY ADDS**

- This study demonstrates that SAD is common in PLWH, even among 22% of non-smokers and IOS measurements, particularly R5–R20 and AX, showed good diagnostic utility, with clinically relevant cut-off values defined.

**HOW THIS STUDY MIGHT AFFECT RESEARCH, PRACTICE OR POLICY**

- SAD appears to be frequent among people living with HIV. In this context, IOS may serve as a supportive method for the clinical assessment of early small airway involvement, alongside standard pulmonary function testing and enable earlier intervention in this high-risk population.

## Introduction

 HIV infection, first identified in the early 1980s, still affects approximately 40 million people worldwide.^1^ By targeting CD4^+^ T-cells, dendritic cells and macrophages, HIV undermines immune competence and drives chronic systemic inflammation.^2 3^ Since widespread antiretroviral therapy has markedly reduced opportunistic infections, non-infectious complications of HIV infection have become more prominent. Specifically, respiratory disorders are now a leading cause of morbidity and mortality in people living with HIV (PLWH).^4^ HIV has been implicated in several chronic airway disorders, including emphysema, chronic obstructive pulmonary disease (COPD) and bronchiectasis, independent of smoking behaviour.^4^,^6^

In many airway diseases, the earliest functional changes occur within the small airways, the bronchioles measuring <2 mm in diameter. Although this region of the lung occupies the largest fraction of the lung’s cross-sectional area, conventional spirometric indices may remain normal in the presence of obstructive diseases of the small airways. Accordingly, it is often termed the lung’s ‘silent zone’, where small airway disease (SAD) can progress undetected until more proximal airways become involved.^7 8^ Small airways are often recognised as major sites of airway inflammation and obstruction, and SAD is common even in mild asthma and all severities of obstructive lung diseases.^9 10^ Therefore, assessment of small airway function is essential, especially in at-risk populations such as PLWH, who are susceptible to chronic airway inflammation.

Impulse oscillometry (IOS) assesses respiratory mechanics by superimposing pressure oscillations at varying frequencies on tidal breathing and measuring the changes in airflow. From these signals, the device calculates respiratory impedance, which consists of the sum of resistance (R) and reactance (X) components that reflect airflow obstruction and the elastic properties of the lung. Lower‐frequency oscillations can reach the entire bronchial tree including lung periphery, whereas higher frequencies mainly sample the central airways. IOS can distinguish small airway obstruction by detecting an increase in peripheral airway resistance and appears to be more sensitive than spirometry for identifying early SAD.^11^ It is effort‐independent, quick to perform and well tolerated, advantages that make it attractive for monitoring vulnerable populations such as PLWH. Early identification of SAD is particularly important in PLWH because chronic airway inflammation may progress silently and cause development of chronic airway diseases if not recognised at an early stage.

The aim of the present study was to evaluate the prevalence and characteristics of SAD in PLWH by comparing demographic, clinical features and IOS parameters. In addition, we aimed to evaluate the diagnostic performance of commonly used IOS parameters in identifying SAD in this population in order to explore their supportive role in the assessment of SAD in PLWH.

## Methods

### Study design and setting

A cross-sectional study was conducted between November 2023 and August 2024 in the Pulmonary Function Laboratory of the Department of Chest Diseases at Istanbul University-Cerrahpasa, Faculty of Medicine.

### Participants

Patients aged ≥18 years who had confirmed HIV infection, who were receiving antiretroviral therapy (ART), and who agreed to participate were included consecutively. To exclude the effects of bronchodilators or acute infections on airway geometry, patients who had a known diagnosis of asthma or COPD, or active pulmonary or laryngeal malignancy, or an active respiratory tract infection were excluded. However, participants who had a forced expiratory volume in 1 s (FEV_1_)/forced vital capacity (FVC) ratio below 0.75 in the current spirometric assessment, but lacked a prior diagnosis of obstructive airway disease, were included to reflect the real-life spectrum of undiagnosed airway impairment in PLWH. Eligible individuals were identified by their physician during routine visits to the Infectious Diseases HIV Outpatient Clinic and were referred to the Pulmonary Function Laboratory.

### Variables and data sources

After verbal and written information was provided by the physician, written informed consent was obtained. Demographic variables (age, sex, body mass index (BMI)), smoking history, biomass exposure, presence of chronic diseases and respiratory symptoms (dyspnoea, cough, sputum production, chest pain) were recorded. The modified Medical Research Council dyspnoea score was documented for those who reported dyspnoea. History of pneumonia was recorded. HIV-related data (date of diagnosis, ART regimen, duration of ART, initial and current CD4 counts, baseline and current HIV RNA levels, any treatment discontinuation and presence of AIDS-defining illnesses) were obtained from medical records.^12^

### Pulmonary function tests

#### Impulse oscillometry

IOS was done using the IOS device (Masterscreen, Jaeger, Würzburg, Germany). Since the inspiratory and expiratory manoeuvres required in spirometry can influence the airway resistance, IOS was done prior to spirometry. The device was calibrated daily before testing in accordance with the standard EU module, and appropriate reference values were selected.^13^ All tests were done by a trained pulmonologist.

Patients were positioned sitting upright with the head in a neutral position. A nose clip was used to occlude the nose, and patients were instructed to breathe quietly through a mouthpiece while gently supporting their cheeks with their hands throughout the measurement. In accordance with American Thoracic Society/European Respiratory Society (ATS/ERS) acceptability criteria, three artefact-free measurements of 20 s each were recorded.^13^

Resistance varies depending on the level of the airway reached by the sound waves; at a frequency of 5 Hz, total airway resistance (R5), which represents the entire airway tree, is measured, while at 20 Hz, resistance reflects only the central airway resistance (R20); small airway resistance is indirectly calculated using the difference between these two values (R5–R20). Reactance (X) reflects the capacitive and inertive properties of the airways; capacitance indicates elasticity and has negative values, whereas inertance represents the tendency to resist changes in flow and has positive values. Since elasticity predominates in the small airways, reactance is negative at lower frequencies (eg, 5 Hz), whereas inertance predominates in the central airways, resulting in positive reactance at higher frequencies (eg, 20 Hz). Resonant frequency (Fres) is the frequency at which total reactance equals zero and capacitance and inertance are equal; although it does not directly assess airway function, an increase in Fres is often a result of more negative low-frequency reactance in obstructive and restrictive diseases, with a normal adult range of 7–12 Hz. The area of reactance (AX) represents the area under the curve (AUC) of negative reactance up to the Fres, reflects lung elastic properties and increases in diseases affecting the peripheral airways or lung parenchyma.^11 13^ IOS is widely used in the assessment of airway resistance, particularly in asthma, where increases in R5 as well as AX and Fres values are observed. Additionally, IOS offers advantages over spirometry in assessing bronchodilator response and bronchial hyperresponsiveness. In asthmatic patients, parameters such as R5, R5–R20 and AX have been found to be more sensitive in evaluating asthma control, quality of life and exacerbations.^14^,^16^ In patients with COPD, increased R5, R5–R20, AX and Fres have been shown to be related to the FEV_1_/FVC ratio, supporting the use of IOS in early disease assessment and follow-up.^17 18^

For each patient, recorded IOS parameters included R5, R20, R5–R20, X5, X20, AX and Fres.

#### Forced spirometry

A spirometer device (Vyntus ONE, Germany) was used for flow–volume measurements. Measurements were obtained after IOS testing, in accordance with the 2019 criteria of the ERS/ATS.^19^ Daily volume calibration was done before tests. All tests were done by a pulmonologist.

Predictive values were automatically calculated by the device based on the patient’s age, BMI, sex and race. The following measurements were recorded: FEV_1_, per cent predicted FEV_₁_ (%FEV_1_), FVC, per cent predicted FVC, FEV_1_/FVC ratio, maximal mid-expiratory flow (MMEF 25–75), per cent predicted MMEF 25–75 (%MMEF 25–75), FEV_3_ and FEV_6_.

A value of MMEF 25–75 below 65% of the predicted value was used to define spirometric SAD, based on the largest population-based study included in the review published by Xiao *et al*.^20^

### Sample size

Sample size calculation was done using OpenEpi.^21^ Assuming an expected prevalence of SAD of 41% among PLWH, with an absolute precision of 10% and a 95% CI, the minimum required sample size for the study was determined to be 93 patients.^20^

### Statistical analysis

SPSS V.25.0 (IBM) was used for statistical analyses. The normality of data distribution was assessed using descriptive statistics and normality tests (Kolmogorov-Smirnov test and Shapiro-Wilk test). Continuous variables with normal distribution were presented as mean±SD, while those without normal distribution were presented as median (25th–75th percentile). For comparisons of normally distributed continuous variables, the Student’s t-test was used, whereas the Mann-Whitney U test was used for non-normally distributed continuous variables. Categorical variables were analysed using the χ^2^ test or Fisher’s exact test, where appropriate. Spearman correlation analysis was used to assess relationships between continuous variables. Correlation coefficients were evaluated as r=0.00–0.199 as very weak, r=0.20–0.399 as weak, r=0.40–0.599 as moderate, r=0.60–0.799 as strong and r=0.80–1.00 as very strong correlation. Receiver operating characteristic (ROC) analysis was used to evaluate the diagnostic performance of IOS measurements in identifying SAD and to determine optimal cut-off values. The value with the highest Youden index was selected as the cut-off. Univariate logistic regression (LR) was performed to assess possible risk factors for SAD. Variables having p<0.20 in univariate analysis were evaluated in multivariate LR (with backward LR). Results were given as OR and 95% CI. A p value of <0.05 was considered statistically significant.

## Results

Between November 2023 and August 2024, all eligible patients were consecutively assessed, yielding 154 individuals for initial screening. 25 were excluded due to known diagnosis of an airway disease and treated with inhaler therapies (n=13), active pulmonary/laryngeal malignancy (n=2) or an active respiratory infection (n=10). A total of 129 patients who met the inclusion criteria were enrolled (online supplemental figure 1). Two patients were excluded due to missing pulmonary function test data. A total of 127 patients were evaluated for the final analysis.

### Demographic and clinical features

The mean age of the 127 included patients was 43.5±12.5 years, and 85.8% were male. A total of 47.2% of the patients had never smoked. AIDS-defining illnesses were present in 19.7% of the patients. The median current CD4 count was 633 (418.5–888.2), and the median baseline HIV RNA level was 125 000 (31 700–5 905 000). A total of 46 patients (36%) had an FEV_1_/FVC ratio ≤0.75. Demographic characteristics and HIV-related data are presented in table 1.

**Table 1:** Demographic data and HIV infection-related data

Age	43.5±12.5
Sex (male) (%)	109 (85.8)
Body mass index (kg/m^2^)	25.7 (23.3–28.9)
Smoking history	
Never	60 (47.2)
Active	51 (40.2)
Ex-smoker	16 (12.6)
Passive exposure	37 (29.4)
Biomass exposure	36 (28.3)
Chronic illness	46 (36.2)
Family history of airway disease	29 (22.8)
History of tuberculosis	4 (3.1)
History of COVID-19	41 (32.3)
History of pneumonia	19 (15)
History of pulmonary embolism	2 (1.6)
Dyspnoea	25 (19.7)
mMRC 0–1	17 (13.4)
mMRC 2	6 (4.7)
mMRC 3	1 (0.8)
mMRC 4	1 (0.8)
Cough	30 (23.6)
Sputum	27 (21.3)
Chest pain	1 (0.8)
AIDS defining disease	25 (19.7)
ART resistance	5 (3.9)
Irregular ART use, cessation of the treatment	16 (12.6)
Initial CD4 count (cell/µL)	301 (130–524.2)
Current CD4 count (cell/µL)	633 (418.5–888.2)
Initial HIV RNA count ((copy/mL)/1000)*	125 (31.7–590.5)
Current HIV RNA count ((copy/mL)/1000)*	0 (0–0)
Duration of seronegativity (months)	3 (3–5)
ART duration (years)	5 (3–8)

Parameters that followed a normal distribution were presented as mean±SD, while those that did not follow a normal distribution were presented as median (25th–75th percentile). Categorical data were presented as counts and percentages.

The minimum and maximum current HIV RNA levels in the groups are 0 and 1 860 507 for the group without small airway disease (SAD), and 0 and 30 665 for the group with SAD, respectively.

* HIV RNA levels are presented as median (min–max values).

ART, antiretroviral treatment; mMRC, modified Medical Research Council.

### Comparison of patients with and without SAD

Spirometric SAD (defined as MMEF value below 65% of predicted) was identified in 34% of all patients and 22% in non-smokers. When comparing patients with and without SAD, age, smoking history (in pack-years) and biomass exposure were found to be significantly greater in the SAD group (p<0.0001, p<0.0001 and 0.0454, respectively). The duration of ART was also significantly longer in the SAD group (p=0.0312). No statistically significant differences were observed between the groups for the remaining variables (table 2).

**Table 2:** Comparison of groups with and without spirometric SAD

	Spirometric SAD (−) (n=84)	Spirometric SAD (+) (n=43)	P value
Demographic data and HIV			
Age	40.3±11.4	49.7±12.3	**<0.0001**
Sex (male)	72 (85.7)	37 (86)	0.9591
Body mass index (BMI)	25.3 (23.4–28.7)	26.4 (22.1–29)	0.9763
History of smoking (pack×years)	0 (0–9.7)	19 (0–30)	**<0.0001**
Biomass exposure	19 (22.6)	17 (39.5)	**0.0454**
Family history of airway disease	16 (19.3)	13 (30.2)	0.1661
AIDS defining disease	13 (15.5)	12 (27.9)	0.0951
Initial CD4 count (cell/µL)	333 (184–540)	239 (39–503)	0.0753
Current CD4 count (cell/µL)	648 (418–857)	621 (414–892)	0.9305
Initial HIV RNA count ((copy/mL)/1000)*	116 (28–650)	167 (39–544)	0.4024
Current HIV RNA count ((copy/mL)/1000)*	0 (0–1861)	0 (0–31)	0.3322
HIV RNA negativity duration (months)	3 (3-5)	4 (3-6)	0.1873
Antiretroviral treatment duration (months)	48 (27–84)	84 (36–120)	**0.0312**
Dyspnoea	15 (17.9)	10 (23.3)	0.4603
Cough	19 (22.6)	11 (25.6)	0.7101
History of pneumonia	10 (11.9)	9 (20.9)	0.1774
Eosinophil count (cell/µL)	100 (100–200)	100 (100–200)	0.9082
Eosinophil percentage	2.3 (1.2–3.05)	1.8 (1.4–2.6)	0.9442
Spirometry parameters			
FEV_1_ (mL)	4071.2±768.9	3058.6±834.5	**<0.0001**
FEV_1_%	108.3±11.6	90.8±17.2	**<0.0001**
FVC (mL)	5127.2±955.7	4455.5±1185.1	**<0.0001**
FVC%	112.1±13.4	107.3±18.5	0.1412
FEV_1_/FVC (%)	79.88 (76.8–83.2)	70.54 (63.9–73.5)	**<0.0001**
FEV_3_ (mL)	4785±1	3815±1.1	**<0.0001**
FEV_6_ (mL)	4930±0.9	4200±1.1	**<0.0001**
FEV_3_/FEV_6_ (%)	95 (94–96)	92 (90–93)	**<0.0001**
FEV_3_/FVC (%)	93 (91–95)	87 (82–90)	**<0.0001**
FEV_6_/FVC (%)	97 (95–98)	94 (91–96)	**<0.0001**
Impulse oscillometry			
R5 (kPa/L/s)	0.4 (0.3–0.5)	0.45 (0.4–0.5)	**0.0041**
R5 (%)	141 (115.3–160.8)	152 (130–185)	**0.0193**
R20 (kPa/L/s)	0.33 (0.3–0.4)	0.37 (0.3–0.4)	**0.0373**
R20 (%)	137 (111–163)	146 (116–178)	0.1142
R5–R20 (kPa/L/s)	0.05 (0–0.1)	0.08 (0.5–0.12)	**0.0001**
X5 (kPa/L)	−0.05 (−0.08–−0.04)	−0.07 (−0.1–−0.04)	0.6801
X20 (kPa/L)	0.05 (0.02–0.07)	0.02 (−0.1–0.05)	**0.0104**
AX (kPa/L)	0.25 (0.12–0.42)	0.55 (0.2–0.9)	**<0.0001**
Fres (Hz)	13.5 (10.3–17.1)	17.6 (13.2–21.5)	**<0.0001**

Parameters that followed a normal distribution were presented as mean±SD, while those that did not follow a normal distribution were presented as median (25th–75th percentile). Categorical data were presented as counts and percentages. Bold values indicate statistically significant results (p < 0.05).

* HIV RNA levels are presented as median (min–max values).

FEV_1_, forced expiratory volume in 1 s; FEV_3_, forced expiratory volume in 3 s; FEV_6_, forced expiratory volume in 6 s; FVC, forced vital capacity; SAD, small airway disease.

When spirometric measurements were compared between the two groups, FEV_1_, %FEV_1_, FVC, FEV_1_/FVC, FEV_3_, FEV_6_, FEV_3_/FVC and FEV_6_/FVC values were found to be significantly lower in the group with SAD (p<0.0001).

When IOS measurements were evaluated, R5, R5%, R20, R5–R20, AX and Fres were significantly higher, while X20 was lower in the SAD group.

In the LR analysis, possible risk factors associated with SAD were evaluated. In the univariate analysis, age, smoking history, biomass exposure and duration of ART were found to be significantly associated with SAD (p<0.0001, p=0.0001, p=0.0484, p=0.0043, respectively). In the multivariate analysis, smoking history, duration of ART and history of pneumonia were identified as independent risk factors for SAD (table 3).

**Table 3:** Small airway disease risk factors

	Univariate LR			Multivariate LR		
	OR	95% CI	P value	OR	95% CI	P value
Age	1.069	1.033 to 1.106	**<0.0001**			
History of smoking	1.052	1.022 to 1.082	**0.0001**	1.065	1.032 to 1.099	**<0.0001**
Biomass exposure	2.237	1.008 to 4.963	**0.0484**			
Family history of airway disease	1.815	0.776 to 4.242	0.1694			
Presence of AIDS defining disease	2.114	0.867 to 5.153	0.1002			
Initial CD4 count (cell/µL)	0.998	0.997 to 1.000	0.0541			
Antiretroviral treatment duration	1.155	1.047 to 1.274	**0.0043**	1.184	1.054 to 1.332	**0.0049**
History of pneumonia	1.959	0.729 to 5.260	0.1822	3.410	1.073 to 10.836	**0.0381**

Parameters having p<0.20 in univariate analysis were evaluated in multivariate logistic regression (with backward LR). Bold values indicate statistically significant results (p < 0.05).

LR, logistic regression.

### Correlation of spirometry and IOS

R5 and R5–R20 values were found to have a moderate negative correlation with FEV_1_, FVC, FEV_3_ and FEV_6_ values. Fres and AX values showed a weak to moderate negative correlation with all spirometric parameters used to assess SAD (table 4).

**Table 4:** Assessment of the relation between spirometry and impulse oscillometry parameters

	R5	R20	R5–R20	X5	X20	Fres	AX
FEV_1_	**−0.45** **(<0.0001)**	**−0.35** **(<0.0001)**	**−0.45** **(<0.0001)**	0.075 (0.4033)	**0.36** **(<0.0001)**	**−0.43** **(<0.0001)**	**−0.46** **(<0.0001)**
FEV_1_%	**−0.23** **(0.0092)**	**−0.19** **(0.0322)**	**−0.30** **(0.0001)**	**0.19** **(0.0274)**	**0.28** **(0.0001)**	**−0.34** **(<0.0001)**	**−0.41** **(<0.0001)**
FVC	**−0.41** **(<0.0001)**	**−0.35** **(<0.0001)**	**−0.43** **(<0.0001)**	0.05 (0.5342)	**0.29** **(0.0001)**	**−0.36** **(<0.0001)**	**−0.41** **(<0.0001)**
FVC%	−0.16 (0.0721)	−0.15 (0.1002)	**−0.26** **(0.0043)**	0.08 (0.3373)	**0.20** **(0.0245)**	**−0.26** **(0.0031)**	**−0.31** **(<0.0001)**
FEV_1_/FVC	−0.17 (0.0591)	−0.78 (0.3835)	**−0.20** **(0.0221)**	0.14 (0.1092)	**0.25** **(0.0041)**	**−0.29** **(0.0011)**	**−0.30** **(0.0001)**
FEV_3_	**−0.44** **(<0.0001)**	**−0.38** **(<0.0001)**	**−0.45** **(<0.0001)**	0.05 (0.5703)	**0.34** **(<0.0001)**	**−0.42** **(<0.0001)**	**−0.44** **(<0.0001)**
FEV_6_	**−0.40** **(<0.0001)**	**−0.37** **(<0.0001)**	**−0.40** **(<0.0001)**	0.04 (0.6352)	**0.30** **(0.0001)**	**−0.36** **(<0.0001)**	**−0.40** **(<0.0001)**
FEV_3_/FVC	**−0.27** **(0.0021)**	**−0.22** **(0.0143)**	**−0.28** **(0.0001)**	0.07 (0.4084)	**0.29** **(0.0001)**	**−0.33** **(<0.0001)**	**−0.30** **(0.0001)**
FEV_6_/FVC	**−0.28** **(0.0031)**	**−0.25** **(0.0072)**	**−0.25** **(0.0081)**	0.08 (0.3734)	0.17 (0.0663)	**−0.26** **(0.0041)**	**−0.26** **(0.0049)**
FEV_3_/FEV_6_	**−0.24** **(0.0091)**	**−0.25** **(0.0074)**	**−0.22** **(0.0161)**	0.05 (0.5582)	**0.22** **(0.0164)**	**−0.26** **(0.0041)**	**−0.27** **(0.0042)**
MMEF 25–75	**−0.35** **(<0.0001)**	**−0.27** **(0.0022)**	**−0.36** **(<0.0001)**	0.13 (0.1612)	**0.33** **(<0.0001)**	**−0.40** **(<0.0001)**	**−0.42** **(<0.0001)**
MMEF 25%–75%	**−0.28** **(0.0001)**	**−0.18** **(0.0461)**	**−0.31** **(<0.0001)**	**0.18** **(0.0343)**	**0.32** **(0.0001)**	**−0.37** **(<0.0001)**	**−0.43** **(<0.0001)**

Results are defined as correlation coefficient (p value). Bold values indicate statistically significant results (p < 0.05).

FEV_1_, forced expiratory volume in 1 s; FEV_3_, forced expiratory volume in 3 s; FEV_6_, forced expiratory volume in 6 s; FVC, forced vital capacity.

### Evaluation of the effect of eosinophil levels and smoking history on airway physiology

No statistically significant correlation was found between serum eosinophil levels and any of the spirometry or IOS values (p>0.05). A weak negative correlation was observed between smoking history (pack-year) and FEV_₁_/FVC (r=−0.37; p<0.0001), FEV_₃_ (r=−0.26; p=0.0031), FEV_₆_ (r=−0.37; p<0.0001), FEV_₆_/FVC (r=−0.37; p<0.0001), MMEF 25–75 (r=−0.32; p=0.0001) and %MMEF 25–75 (r=−0.36; p<0.0001) values.

### Diagnostic performance of IOS parameters in SAD

After defining SAD as a per cent predicted MMEF 25–75 value <65% of predicted as measured by spirometry, an ROC analysis was done to evaluate performance characteristics for IOS measurements. The ROC curves indicating the discriminative accuracy of IOS measurements for SAD are presented in online supplemental figure 2. All measurements, except X5, were able to distinguish SAD at a statistically significant level (AUC for X5=0.559, p=0.0694). For R5, the cut-off value was determined to be 0.45 kPa/L/s, with 55.8% sensitivity and 70.2% specificity (AUC=0.655, p=0.0042). For R5–R20, the cut-off was 0.08 kPa/L/s, with 58.1% sensitivity and 71.4% specificity (AUC=0.682, p=0.0001). Among the IOS parameters, AX demonstrated the highest specificity, with a cut-off value of 0.55 kPa/L for distinguishing SAD (AUC=0.727, sensitivity=51.2%, specificity=91.7%, p<0.0001) (table 5).

**Table 5:** Cut-off values and test performance of impulse oscillometry parameters in discriminating small airway disease

Parameters	Cut-off value	AUC	Sensitivity (%)	Specificity (%)	LR (+)	LR (−)	Youden index	P value
R5 (kPa/L/s)	0.45	0.655	55.8	70.2	1.88	0.63	0.26	0.0042
R20 (kPa/L/s)	0.39	0.614	48.8	73.8	1.86	0.69	0.23	0.0373
R5–R20 (kPa/L/s)	0.08	0.682	58.1	71.4	2.03	0.59	0.30	**0.0001**
FRES (Hz)	18	0.694	48.8	86.9	3.73	0.59	0.36	**<0.0001**
AX (kPa/L)	0.55	0.727	51.2	91.7	6.14	0.53	0.43	**<0.0001**
X20 (kPa/L)	0.03	0.676	62.8	70.2	2.11	0.53	0.33	**0.0001**

Bold values indicate statistically significant results (p < 0.05).

AUC, area under curve; LR, likelihood ratio.

## Discussion

This study demonstrates that SAD is detected at a considerable frequency among PWLH and that IOS can provide supportive information about small airway involvement. AX was found to be the most sensitive measurement for detecting SAD in this population, while R5 and R5–R20 were also useful. Smoking history, duration of ART and history of pneumonia were identified as independent risk factors for SAD.

SAD was first described by Detels *et al* in 1979, using an MMEF 25%–75% predicted value below 75% as the diagnostic criterion in a study of nearly 8000 individuals.^22^ A systematic review examined 25 studies and reported varying MMEF 25–75 cut-off values ranging from 60% to 75%. Due to methodological differences and heterogeneity in disease prevalence, there is no consensus on the optimal threshold.^23^ In our study, spirometric SAD was defined using a %MMEF 25–75 value below 65%, based on the most recent and largest population-based study included in the same review.^20^ The prevalence of spirometric SAD among the PLWH included was 34%. In a previous study conducted in Africa involving 104 HIV-infected children, the frequency of abnormal MMEF 25–75 was found to be 52%; most of the patients in that study lived in rural areas.^24^ Various factors, such as environmental exposures in that region and the clinical course of HIV infection, may have contributed to the difference in prevalence.

SAD has been reported at substantial rates in the general population, providing an important reference for interpreting our findings. A large study from China reported an overall prevalence of 43.5%.^20^ In asthma, SAD prevalence is reported as 50%–60% across all disease severities, and in individuals who are at high risk for COPD, the prevalence is reported as 54.1%.^25 26^ When these results are compared with our findings, the SAD prevalence of 34% represents a health problem in PLWH that should not be underestimated.

Additional evidence supports small airway involvement in HIV. Studies have shown that SAD is more frequent in non-smoking PLWH than in HIV-negative controls, and more than 40% of perinatally infected adolescents exhibit symptoms of SAD.^27 28^ Although early initiation of ART is associated with better pulmonary function in perinatally exposed children, the risk of small airway abnormalities appears to persist into adulthood.^29^

Our findings showed that age, smoking history and biomass exposure were associated with SAD. These determinants are in line with those identified in the previous literature, which has proposed that SAD has similar risk factors with COPD and may represent an early indicator of obstructive airway diseases.^20 30^

In our study, smoking exposure, duration of antiretroviral therapy and history of pneumonia were identified as independent risk factors for the development of SAD. Age was included in the multivariate regression model, and duration of ART showed an independent association with SAD after adjustment. These findings suggest that multiple mechanisms may contribute to chronic airway injury in people who live with HIV. Chronic inflammation caused by recurrent respiratory infections and direct airway involvement in individuals with a history of pneumonia may result in lasting structural damage. In the literature, ART has been shown to increase CD4 T lymphocyte counts, but it may also trigger inflammatory responses such as sarcoidosis through immune reconstitution syndrome in the early phase.^31^ The association between longer ART duration and SAD in our study supports the hypothesis that such inflammation may lead to microscopic fibrotic changes in the bronchioles over time. These findings highlight the need for comprehensive respiratory follow-up in PLWH, including early detection of airway disease, management of infection-related damage and support for smoking cessation.

In our study, the significantly higher values of R5, R20, R5–R20, AX and Fres in the group with spirometric SAD are consistent with findings in the literature.^11 14 23 32^ In a study by Winkler *et al*, an R5 value below 130%–150% of the predicted value was proposed as normal.^33^ Although this is not a validated threshold, we found that even in the group without SAD, the mean R5% was higher than 140%, and it was significantly higher in the SAD group. Similarly, Desiraju and Agrawal reported a normal Fres range of 7–12 Hz in adults.^11^ In our study, the mean Fres value in the group without SAD was 13.5 Hz, and it was significantly higher in the SAD group. These findings suggest that even when routine spirometry does not indicate SAD, there may still be airway pathology in PLWH, and elevated R5% and Fres values may signal a risk for future airway disease.

In this study, no statistically significant correlation was found between HIV-related parameters (CD4 counts, HIV RNA levels and duration of ART) and IOS findings. This may be due to the fact that IOS measures small airway resistance at the mechanical level, which may not directly reflect systemic immunological or virological status. Additionally, most patients in our study had well-controlled HIV infection, which could have reduced variability and limited the ability to detect such associations.

Peripheral eosinophilia is commonly observed in the type 2 inflammation phenotype of asthma, and elevated serum eosinophil levels are associated with asthma severity and exacerbation frequency.^34^ In patients who have COPD, eosinophil counts have also been linked to exacerbation severity and reduced lung function.^35^ In our study, the absence of a correlation between serum eosinophil levels and pulmonary function test measurements may reflect the effects of immune suppression in PLWH. Alternatively, it may suggest that airway pathology in HIV differs from that seen in asthma or certain COPD endotypes and may not primarily involve eosinophilic inflammation. Future studies assessing eosinophilic airway inflammation through fractional exhaled nitric oxide (FeNO) measurement or sputum eosinophilia may help clarify the mechanisms underlying airway disease in HIV.

This study also evaluated the diagnostic performance of IOS measurements in detecting SAD in PLWH. A specificity of 91.7% for AX suggests that this measurement may substantially reduce false positives in SAD diagnosis. R5 and R5–R20 were found to have a moderate ability to distinguish SAD, with areas under the curve of 0.655 and 0.682, respectively. Although IOS parameters such as R5–R20 and AX are frequently used in research to describe SAD, their diagnostic performance remains limited. Reported moderate diagnostic performance indicates that these cut-off values cannot serve as a reliable stand-alone screening method for SAD. Therefore, these parameters should be interpreted cautiously as supportive physiological information with the clinical assessment. Fres, although not directly linked to a specific mechanical property of the lungs, reflects the transition between low-frequency capacitance and high-frequency inertance.^19^ The 18 Hz cut-off value identified in our study, which is higher than values typically reported in the literature, may indicate more prominent peripheral airway pathology in HIV-infected individuals. Furthermore, the high specificity of Fres (86.9%) reinforces its potential diagnostic role, alongside AX, in identifying SAD.

This study has limitations. Due to its cross-sectional design, it was not possible to assess the causal relationship between HIV infection and SAD or to evaluate the impact of dynamic changes in CD4 counts and viral load on pulmonary function. Since this study did not include an external validation cohort, the generalisability of the findings may be limited. In addition, patients who have reduced FEV_₁_/FVC ratio were not excluded if they had no formal diagnosis of airway disease to reflect the real-life spectrum of undiagnosed airway impairment in PLWH. Therefore, the observed IOS abnormalities may reflect both SAD and coexisting proximal airway involvement. Long-term follow-up studies or stratified analyses including isolated SAD may provide additional insight. Additionally, Z scores were not used in the interpretation of spirometry because they were not yet implemented or routinely recorded in our clinical practice when the data collection began. This may have limited our ability to reflect fully on spirometric assessment with current guideline recommendations.

Most participants in our study were male, reflecting the demographic distribution of HIV-infected individuals in our centre. However, sex imbalance may limit the generalisability of our findings to female patients, who may exhibit airway involvement or disease progression in different patterns. Future studies with more balanced sex representation are needed to evaluate possible sex-specific differences in small airway physiology.

This study highlights the clinical relevance of SAD, a frequently overlooked but common condition among PLWH, and is the first to evaluate this population using IOS. By capturing early functional changes in the peripheral airways, IOS may help identify patients at risk before spirometric abnormalities emerge. These findings suggest a potential role for IOS in the early detection, prevention and monitoring of airway diseases in this vulnerable population.

## Conclusion

SAD is a common health concern among PLWH. IOS may serve as a supportive tool for the clinical assessment of small airway involvement. The findings of this study suggest that IOS can contribute to the evaluation of early detection of small airway pathology in HIV, potentially helping to prevent and manage more severe airway diseases, but its findings should be interpreted together with clinical and spirometric data rather than as a stand-alone diagnostic tool.

## Supplementary material

10.1136/bmjresp-2025-003719 online supplemental figure 1

10.1136/bmjresp-2025-003719 online supplemental figure 2

## Data Availability

Data are available upon reasonable request.

## References

[1] (2024). Global 2024 AIDS update/UNAIDS data. https://www.unaids.org/sites/default/files/media_asset/2024-unaids-global-aids-update.

[2] Robb ML, Ananworanich J (2016). Lessons from acute HIV infection. Curr Opin HIV AIDS.

[3] Nyamweya S, Hegedus A, Jaye A (2013). Comparing HIV-1 and HIV-2 infection: Lessons for viral immunopathogenesis. Rev Med Virol.

[4] Fitzpatrick ME, Kunisaki KM, Morris A (2018). Pulmonary disease in HIV-infected adults in the era of antiretroviral therapy. AIDS.

[5] Attia EF, Miller RF, Ferrand RA (2017). Bronchiectasis and other chronic lung diseases in adolescents living with HIV. Curr Opin Infect Dis.

[6] Petrache I, Diab K, Knox KS (2008). HIV-associated pulmonary emphysema: a review of the literature and inquiry into its mechanism. Thorax.

[7] Carr TF, Altisheh R, Zitt M (2017). Small airways disease and severe asthma. World Allergy Organ J.

[8] Burgel P-R, Bergeron A, de Blic J (2013). Small airways diseases, excluding asthma and COPD: an overview. Eur Respir Rev.

[9] Galant SP, Kuks PJM, Kole TM (2025). Assessment of the role of small airway dysfunction in relation to exacerbation risk in patients with well controlled asthma (ATLANTIS): an observational study. Lancet Respir Med.

[10] Vardaloglu I, Sousa-Pinto B, Bousquet J (2024). In symptomatic patients on as-needed inhaled corticosteroids-formoterol, VAS asthma is associated with small airways resistance. J Asthma.

[11] Desiraju K, Agrawal A (2016). Impulse oscillometry: The state-of-art for lung function testing. Lung India.

[12] AIDS-defining conditions. https://www.cdc.gov/mmwr/preview/mmwrhtml/rr5710a2.htm.

[13] King GG, Bates J, Berger KI (2020). Technical standards for respiratory oscillometry. Eur Respir J.

[14] Bednarek M, Grabicki M, Piorunek T (2020). Current place of impulse oscillometry in the assessment of pulmonary diseases. Respir Med.

[15] Yang X, Liu M, Shi H (2025). Detection of Inadequately Controlled Asthma in Adults Using Impulse Oscillometry and Fractional Exhaled Nitric Oxide. J Asthma Allergy.

[16] Galant SP, Cottini M, Berti A (2025). Small Airway Dysfunction Is an Independent Exacerbation Risk Biomarker in the Mild, Well-Controlled Patient With Asthma: A Frequently Unrecognized High-Risk Phenotype. J Allergy Clin Immunol Pract.

[17] Peng J, Li X, Zhou H (2025). Clinical Value of Impulse Oscillometry in Chronic Obstructive Pulmonary Disease: A Systematic Review and Meta-Analysis. Respiration.

[18] Liwsrisakun C, Chaiwong W, Deesomchok A (2025). The Role of Impulse Oscillometry in Detection of Preserved Ratio Impaired Spirometry (PRISm). Adv Respir Med.

[19] Graham BL, Steenbruggen I, Miller MR (2019). Standardization of Spirometry 2019 Update. An Official American Thoracic Society and European Respiratory Society Technical Statement. Am J Respir Crit Care Med.

[20] Xiao D, Chen Z, Wu S (2020). Prevalence and risk factors of small airway dysfunction, and association with smoking, in China: findings from a national cross-sectional study. Lancet Respir Med.

[21] Dean AG, Sullivan KM, Soe MM OpenEpi: open source epidemiologic statistics for public health. www.OpenEpi.com.

[22] Detels R, Rokaw SN, Coulson AH (1979). The UCLA population studies of chronic obstructive respiratory disease. I. Methodology and comparison of lung function in areas of high and low pollution. Am J Epidemiol.

[23] Knox-Brown B, Mulhern O, Feary J (2022). Spirometry parameters used to define small airways obstruction in population-based studies: systematic review. Respir Res.

[24] Ayuk AC, Ndukwu CI, Uwaezuoke SN (2021). Small-airway disease and its reversibility in human immunodeficiency virus-infected children on highly active antiretroviral therapy: A cross-sectional study in an African setting. Ann Thorac Med.

[25] Usmani OS, Singh D, Spinola M (2016). The prevalence of small airways disease in adult asthma: A systematic literature review. Respir Med.

[26] Wen G, Meng J, Xu Y (2025). Prevalence and Risk Factors of Spirometry-Defined Small Airway Dysfunction in the High-Risk Population for COPD in Yunnan Province, China: A Population Based Cross-Sectional Study. Int J Chron Obstruct Pulmon Dis.

[27] Ronit A, Mathiesen IH, Gelpi M (2017). Small airway dysfunction in well-treated never-smoking HIV-infected individuals. Eur Respir J.

[28] Ferrand RA, Desai SR, Hopkins C (2012). Chronic lung disease in adolescents with delayed diagnosis of vertically acquired HIV infection. Clin Infect Dis.

[29] Collini P (2021). Lung function declines more rapidly in treated HIV-positive people than in HIV-negative people. Lancet Healthy Longev.

[30] Knox-Brown B, Patel J, Potts J (2023). Small airways obstruction and its risk factors in the Burden of Obstructive Lung Disease (BOLD) study: a multinational cross-sectional study. Lancet Glob Health.

[31] Trevenzoli M, Cattelan AM, Marino F (2003). Sarcoidosis and HIV infection: a case report and a review of the literature. Postgrad Med J.

[32] Gong SG, Yang WL, Zheng W (2014). Evaluation of respiratory impedance in patients with chronic obstructive pulmonary disease by an impulse oscillation system. Mol Med Rep.

[33] Winkler J, Hagert-Winkler A, Wirtz H (2009). Die Impulsoszillometrie in der Schweregraddiagnostik obstruktiver Lungenerkrankungen. Pneumologie.

[34] Solomon Y, Malkamu B, Berhan A (2023). Peripheral blood eosinophilia in adult asthmatic patients and its association with the severity of asthma. BMC Pulm Med.

[35] Tashkin DP, Wechsler ME (2018). Role of eosinophils in airway inflammation of chronic obstructive pulmonary disease. Int J Chron Obstruct Pulmon Dis.

