# Supplementary figures and images for: Characteristics of small airway disease in patients with HIV infection: insights from spirometry and impulse oscillometry
Source: BMJ Open Respir Res. 2026 Apr 15;13(1):e003719. doi: 10.1136/bmjresp-2025-003719 (PMC13084963; doi:10.1136/bmjresp-2025-003719)

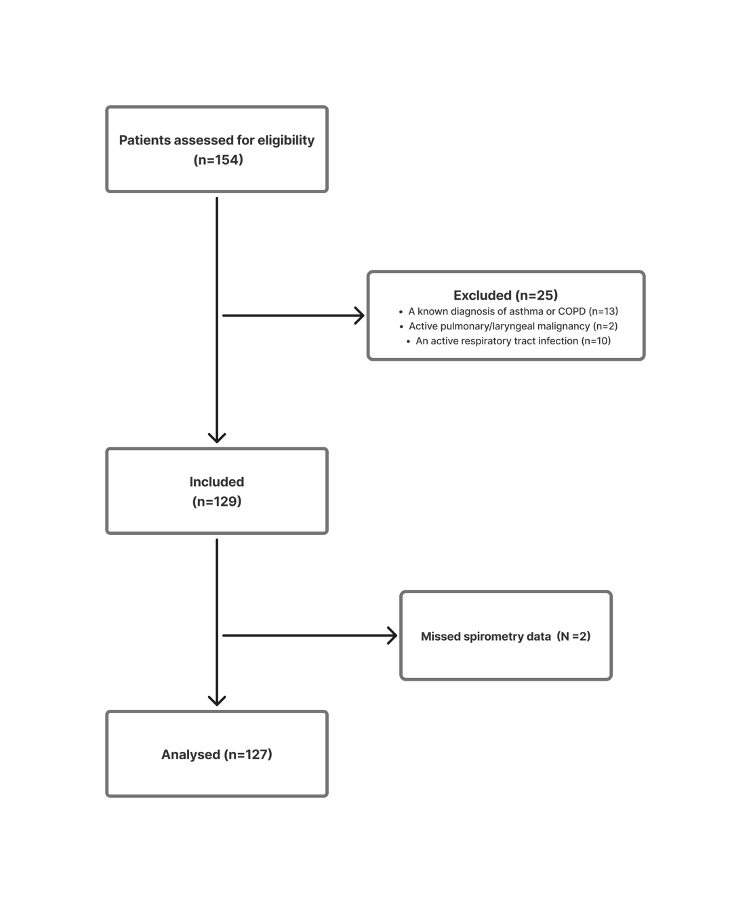

Supplement: online supplemental figure 1 [file bmjresp-13-1-s001.png]

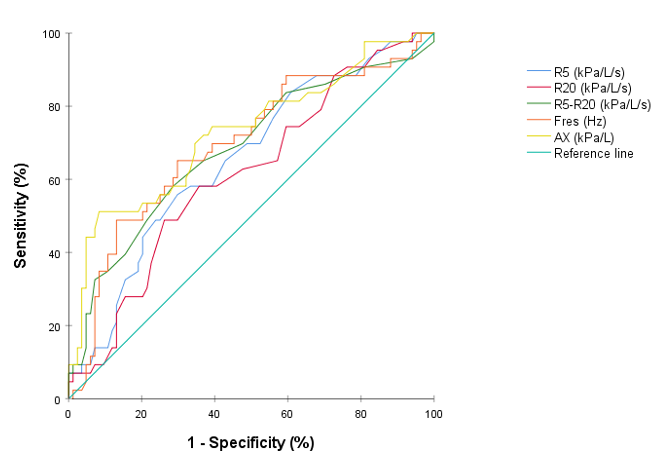

Supplement: online supplemental figure 2 [file bmjresp-13-1-s002.png]
